# Modeling and Control of a Micro AUV: Objects Follower Approach

**DOI:** 10.3390/s18082574

**Published:** 2018-08-06

**Authors:** Jesus Arturo Monroy-Anieva, Cyril Rouviere, Eduardo Campos-Mercado, Tomas Salgado-Jimenez, Luis Govinda Garcia-Valdovinos

**Affiliations:** 1 Energy Division, Center for Engineering and Industrial Development-CIDESI, Santiago de Queretaro, Queretaro 76125, Mexico; tsalgado@cidesi.edu.mx (T.S.-J.); ggarcia@cidesi.edu.mx (L.G.G.-V.); 2 Automation and Control Department, Universidad Politecnica de Tulancingo-UPT, Tulancingo 43629, Hidalgo, Mexico; 3 Ecole Nationale Supérieure de Techniques Avancées-ENSTA, Bretagne, Brest 29806, France; pi@cyril-rouviere.fr; 4 CONACYT, Universidad del Istmo de Tehuantepec-UNISTMO, Tehuantepec 70760, Oaxaca, Mexico; ecampos@conacyt.mx

**Keywords:** submarine robot, modeling, control, embedded system, micro vehicle, nonlinear PD, stability, computer vision

## Abstract

This work describes the modeling, control and development of a low cost Micro Autonomous Underwater Vehicle (μ-AUV), named AR2D2. The main objective of this work is to make the vehicle to detect and follow an object with defined color by means of the readings of a depth sensor and the information provided by an artificial vision system. A nonlinear PD (Proportional-Derivative) controller is implemented on the vehicle in order to stabilize the heave and surge movements. A formal stability proof of the closed-loop system using Lyapunov’s theory is given. Furthermore, the performance of the μ-AUV is validated through numerical simulations in MatLab and real-time experiments.

## 1. Introduction

**Figure 1:**
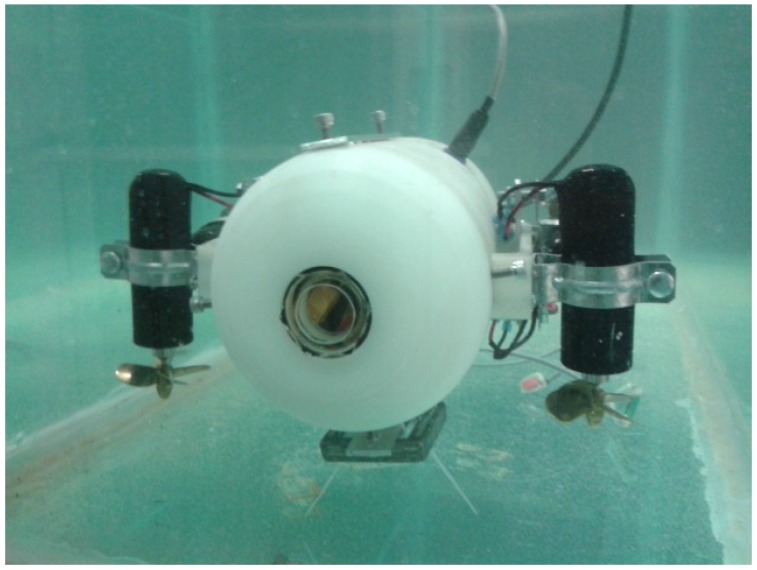
Experimental prototype AR2D2.

Nowadays, the development of autonomous underwater systems is a scientific research topic which deserves a great interest from the international community. There are a huge number of possible applications wherein autonomous underwater vehicles can be used to avoid risky tasks for the human beings. The proper operation of autonomous vehicles requires the convergence of several disciplines among which the automatic control discipline may play an important role when dealing with complex nonlinear dynamics as in the case of AUVs (Autonomous Underwater Vehicles) [1]. The AUVs have traditionally been used for oceanographic research, where these vehicles have been used for mapping and monitoring specific areas; for example, 3D reefs reconstruction, marine cartography, ecosystem exploration, pipeline and structures inspection, etc. [2]. There are other water-based applications which take advantage of the use of AUVs, such as the monitoring of nuclear storage ponds, waste water treatment facilities and archaeology enclosures. These environments differ from the ocean in terms of scale, thus they can be considered as closed spaces [3]. Traditional AUVs have tended to be large scale (meters in length) and high cost, making them unsuitable for small scale environments. One interesting topic from the automatic control point of view is that very often the vertical and horizontal movements are coupled, i.e., forward momentum is required for the AUV to move in the vertical plane. This means that traditional AUV designs are unsuitable for being used as small-scale sensor platforms [4,5,6]. The AR2D2 prototype (see Figure 1) has major features, such as its size and maneuverability compared to traditional vehicles.

The μ-AUV prototype has the shape of a torpedo with the following dimensions: 35 cm × 20 cm front and 30 cm long. Its weight is less than 5 kg, thus this vehicle is considered like a micro AUV [7]. The different AUV’s degrees of freedom are actuated by four thrusters. The geometric shape of the prototype is such that the vertical and horizontal movements can be considered decoupled.

This paper provides details about the platform development and real-time test of a control strategy for heave and surge movements to follow objects. The effectiveness of the proposed control strategy is validated through numerical simulations as well as real-time experiments. Section 2 presents further details on the design and capabilities of the μ-AUV. In Section 3, we briefly describe the dynamic model of the vehicle, whilst Section 4 shows the control strategy for the depth and forward motion through a nonlinear PD controller. Section 5 details the computer vision algorithm developed in order to detect and follow objects, whilst Section 6 presents the simulation of the control law chosen for depth and forward movement, and the experimental results. Finally, concluding remarks and further work proposals are given in Section 7.

## 2. Prototype Description

### 2.1. Embedded System

**Figure 2:**
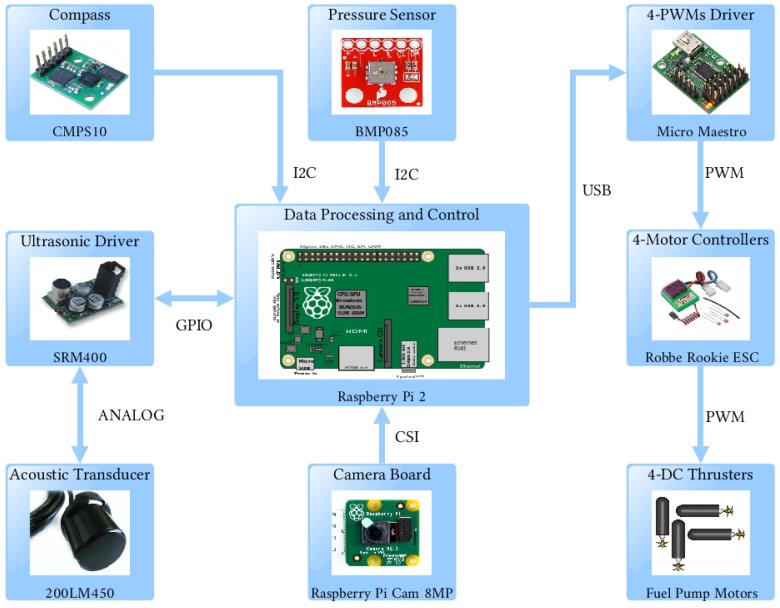
Electronic architecture of the embedded system.

The hardware architecture of the prototype consists of an embedded system that includes a Raspberry Pi 2B, which has a quad-core ARM (Advanced RISC Machines) Cortex-A7 CPU of 900 MHz, 1 GB RAM memory, camera interface (CSI), display interface (DSI), four USB ports, Ethernet port, Full HDMI port, 40 GPIO pins, and a slot to use a micro SD card for an operative system based on a Linux platform that allows for programming the Middleware. This embedded system also includes a compass sensor (CMPS10), an ultrasonic sensor with the driver SRM400, a depth sensor (BMP085) and a Raspberry Pi camera for artificial vision. The Raspberry processes the information from the sensors, computes de control laws and sends signals to the actuators (DC motors) controlled by Pulse Width Modulation using a micro maestro device (six-channel USB) in order to manage four thrusters through the Robbe Rookie 25A ESC (Electronic Speed Control) motor drivers. Real-time communication is provided using an Ethernet LAN interface. In Figure 2, a schematic shows components of the vehicle’s hardware and their interactions.

### 2.2. Computer Vision

The GNSS (Global Navigation Satellite System) signals cannot go through water, acoustic communication throughput is limited, odometry is impossible. However, vision is available. The camera provides rich data about the environment. Moreover, in the case of a Remote Operated Vehicle (ROV), it shows to the the user a lot of information to help him drive the robot. Computer Vision is a way to process these data autonomously, in order to implement obstacle avoidance, object following recognition and following, localization and mapping, without any human intervention.

However, as other sensors, underwater vision poses some particular issues to be solved with respect to usual air vision. The main problem is about picture quality, depending on the water (clearness, suspended particles, etc.), the camera will be able to see less or more details. These details are the key to success for computer vision algorithms, especially dealing with features-based vision. Moreover, many features that the camera will detect are not reliable because they are not static (usually, particles floating in water, bubbles, etc.) Thus, we cannot rely on stereo-vision, optical flow and common mapping, which need a strong feature recognition.

Sometimes, pictures quality is so bad that even a human is not able to use it. Colors often do not have enough contrasts, which does not help to detect and differentiate objects that are not close enough to the robot. Finally, the camera can be reliable only as a low-range sensor, which is not adapted for global navigation.

In fact, underwater vision is problematic only if it tries to mimic air vision. Indeed, what is seen as an issue can be turned to our advantage to have better vision quality than expected before.

Classic computer vision complains about the lack of details under water. However, it then has to filter all of these data to select only what is interesting. With an underwater vision point of view, this heavy filtering is not necessary: it is already done by the environment. Only three colors are visible in water: red, orange and yellow; all others appear as blue. This is why objects we want to detect usually have these colors. Thus, to detect them, color filtering is enough (shape and geometry does not matter).

To get around the problem of low contrast level, HSV (Hue–Saturation–Value) filtering can give good results. A lamp also helps to keep the same luminosity exposition (and avoid moving shadows). It is also a way to measure distance to the object: the better luminosity and contrast are, the closer it is.

### 2.3. Prototype’s Movement Description

**Figure 3:**
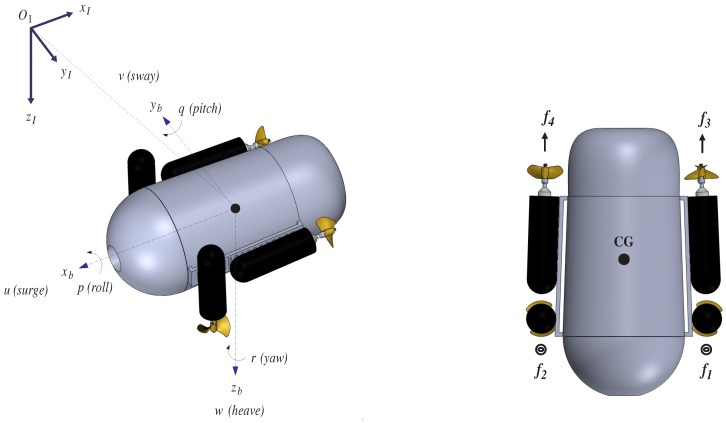
The AR2D2 μAUV, with the body fixed frame $\left(O_{b},x_{b},y_{b},z_{b}\right)$, the earth-fixed frame $\left(O_{I},x_{I},y_{I},z_{I}\right)$, and the forces generated by its four thrusters.

The AR2D2 micro submarine has been designed and built, which is shown in Figure 3, with its body fixed frame $\left(O_{b},x_{b},y_{b},z_{b}\right)$. The center $\left(O_{b}\right)$ of this frame corresponds to the vehicle’s center of gravity, and its axes are aligned with the main axes of vehicle’s symmetry. The movement in the horizontal plane is referred to as surge (along $x_{b}$ axis) and sway (along $y_{b}$ axis), while heave represents the vertical motion (along $z_{b}$ axis). Roll, pitch, and yaw, denoted (ϕ,θ,ψ), are the Euler angles describing the orientation of the vehicle’s body fixed frame with respect to the earth-fixed frame $\left(O_{I},x_{I},y_{I},z_{I}\right)$ [8], while (x,y,z) denote the coordinates of the body-fixed frame center in the earth fixed frame. The propulsion system consists of four thrusters that generate the rotational and translational motion. Concerning the rotational movement of this prototype, roll motion is performed through differential speed control of thrusters 1 and 2. In the same fashion, yaw motion is obtained using thrusters 3 and 4; finally, pitch motion is unactuated with respect to the translational movement of the *z* axis being regulated by decreasing or increasing the combined speed of thrusters 1 and 2. In the same way, the translational movements along the $x_{b}$- and $y_{b}$-axes are obtained by using thrusters 3 and 4 and by controlling the yaw angle.

## 3. Dynamic Model

The dynamics of the vehicle that are expressed in the body-fixed frame can be written in a vectorial setting according to [8]:

(1) $$\begin{array}{c} M\dot{\nu }+C\left(\nu \right)\nu +D\left(\nu \right)\nu +g\left(\eta \right)=\tau +w_{e}, \end{array}$$

(2) $$\begin{array}{c} \dot{\eta }=J\left(\eta \right)\nu , \end{array}$$

where $M\in R^{6\times 6}$ is the inertial matrix, $C\left(\nu \right)\in R^{6\times 6}$ defines the Coriolis-centripetal matrix, $D\left(\nu \right)\in R^{6\times 6}$ represents the hydrodynamic damping matrix, $g\left(\eta \right)\in R^{6\times 1}$ describes the vector of gravitational/buoyancy forces and moments, $\tau ={\left[\tau_{1},\tau_{2}\right]}^{T}={\left[\left[X,Y,Z\right],\left[K,M,N\right]\right]}^{T}\in R^{6\times 1}$ defines the vector of control inputs; $w_{e}\in R^{6\times 1}$ defines the vector of disturbances; $\nu ={\left[\nu_{1},\nu_{2}\right]}^{T}={\left[\left[u,v,w\right],\left[p,q,r\right]\right]}^{T}\in R^{6\times 1}$ denotes the linear and angular velocity vector in the body-fixed frame; $\eta ={\left[\eta_{1},\;\eta_{2}\right]}^{T}={\left[\left[x,y,z\right],\left[\phi ,\theta ,\psi \right]\right]}^{T}\in R^{6\times 1}$ is the position and attitude vector decomposed in the earth-fixed frame, and $J\left(\eta \right)\in R^{6\times 6}$ is the transformation matrix between body frame and earth-fixed frame (for more details, see [9,10]).

### 3.1. Gravity/Buoyancy Forces and Torques

According to Archimedes’s principle, the buoyant force $f_{B}$ applied in the center of buoyancy, which acts on the opposite direction of vehicle weight $f_{W},$ is expressed as follows:

(3) $$f_{B}=-\left[\begin{array}{c} 0\\ 0\\ \rho g\nabla \end{array}\right]\;f_{W}=\left[\begin{array}{c} 0\\ 0\\ mg \end{array}\right],$$

where ρ represents the fluid density, *g* the gravitational acceleration, ∇ the displaced fluid volume and *m* the mass of the vehicle. Now, considering that W=mg and B=ρg∇ by using the zyx-convention for navigation and control application [11], the transformation matrix $J_{1}\left(\eta_{2}\right)=R_{z,\psi }^{T}R_{y,\theta }^{T}R_{x,\phi }^{T}$ is applied in order to obtain the weight and buoyancy forces respect to the body fixed coordinates system:

(4) $$F_{B}=J_{1}{\left(\eta_{2}\right)}^{-1}f_{B}\;F_{W}=J_{1}{\left(\eta_{2}\right)}^{-1}f_{W};$$

consequently,

(5) $$F_{B}=\left[\begin{array}{c} Bsin(\theta )\\ -Bcos(\theta )sin(\phi )\\ -Bcos(\theta )cos(\phi ) \end{array}\right]\;F_{W}=\left[\begin{array}{c} -Wsin(\theta )\\ Wcos(\theta )sin(\phi )\\ Wcos(\theta )cos(\phi ) \end{array}\right].$$

Thus, the restoring forces acting on the vehicle are $f_{g}$=$F_{B}$+$F_{W}$, this is:

(6) $$\begin{array}{c} f_{g} \end{array}=\left[\begin{array}{c} \left(B-W)sin(\theta \right)\\ \left(W-B)cos(\theta )sin(\phi \right)\\ \left(W-B)cos(\theta )cos(\phi \right) \end{array}\right].$$

On the other hand, the restoring moments are described by the following equation:

(7) $$m_{g}=r_{w}\times F_{W}+r_{b}\times F_{B},$$

where $r_{w}={\left[x_{w},y_{w},z_{w}\right]}^{T}$ and $r_{b}={\left[x_{b},y_{b},z_{b}\right]}^{T}$ represent the positions of the center of gravity (CG) and the center of buoyancy (CB), respectively. Based on the design of the vehicle and in order to reduce further analysis, the origin of the body fixed frame is chosen in the gravity’s center; this implies that $r_{w}={\left[0,0,0\right]}^{T}$, while the center of buoyancy is $r_{b}={\left[0,0,-z_{b}\right]}^{T}$. For practical purposes, the buoyancy force is greater than the weight, i.e., $W-B=-f_{b}$. Notice that $f_{b}$ should be smaller than the force produced by the thrusters. Then, from Equations (6) and (7), we have:

(8) $$g\left(\eta \right)=\left[\begin{array}{c} f_{g}\\ m_{g} \end{array}\right]=\left[\begin{array}{c} f_{b}sin\left(\theta \right)\\ -f_{b}cos\left(\theta \right)sin\left(\phi \right)\\ -f_{b}cos\left(\theta \right)cos\left(\phi \right)\\ -z_{b}Bcos\left(\theta \right)sin\left(\phi \right)\\ -z_{b}Bsin\left(\theta \right)\\ 0 \end{array}\right].$$

### 3.2. Forces and Torques Generated by the Thrusters

Figure 3 shows the forces generated by the thrusters acting on the micro submarine. These are described relative to the body-fixed coordinate system as:

$$\hat{f_{1}}=\left[\begin{array}{c} 0\\ 0\\ f_{1} \end{array}\right]\;;\hat{f_{2}}=\left[\begin{array}{c} 0\\ 0\\ f_{2} \end{array}\right]\;;\hat{f_{3}}=\left[\begin{array}{c} f_{3}\\ 0\\ 0 \end{array}\right]\;;\hat{f_{4}}=\left[\begin{array}{c} f_{4}\\ 0\\ 0 \end{array}\right].$$

Summarizing and using the notation of [12], it follows that:

(9) $$\tau_{1}=\left[\begin{array}{c} X\\ Y\\ Z \end{array}\right]=\left[\begin{array}{c} f_{3}+f_{4}\\ 0\\ f_{1}+f_{2} \end{array}\right]$$

and the body-fixed torques generated by the above forces are defined as:

(10) $$\tau_{2}=\sum_{i=1}^{4}l_{i}\times \hat{f_{i}},$$

where $l_{i}=\left(l_{ix},l_{iy},l_{iz}\right)$ is the position vector of the force $\hat{f_{i}}$∀i=1,..,4, with respect to the body-fixed reference frame. Then, the torques generated by the thrusters are described as:

(11) $$\tau_{2}=\left[\begin{array}{c} K\\ M\\ N \end{array}\right]=\left[\begin{array}{c} l_{1y}f_{1}+l_{2y}f_{2}\\ l_{1x}f_{1}+l_{2x}f_{2}\\ l_{3y}f_{3}+l_{4y}f_{4} \end{array}\right].$$

Then,

(12) $$\tau =\left[\begin{array}{c} f_{3}+f_{4}\\ 0\\ f_{1}+f_{2}\\ l_{1y}f_{1}+l_{2y}f_{2}\\ l_{1x}f_{1}+l_{2x}f_{2}\\ l_{3y}f_{3}+l_{4y}f_{4} \end{array}\right].$$

## 4. Control Strategy

For the design of the controller, it is common to assume that the hydrodynamic parameters involved in the dynamical model of the underwater vehicle are unknown. Indeed, they depend on effects and properties that are hard to model or estimate, like added mass, skin friction, vortex shedding, fluid characteristics, etc. Therefore, we propose using a nonlinear PD controller [13].

u(t) is a PD controller, which is depicted as the following equation:

(13) $$u\left(t\right)=K_{P}e\left(t\right)+K_{D}\frac{de(t)}{dt},$$

where e(t) = r(t)−y(t) is the error, r(t) represents the reference, y(t) is the output measurement, and ($K_{P}$, $K_{D}$) are the proportional and derivative gains. In Equation (13), we can notice that, if e(t)→∞, then u(t)→∞; this could lead to system oscillations or in other case saturation of the actuators. In order to prevent damage on the actuators, we propose using a saturation function in each term of Equation (13).

**Figure 4:**
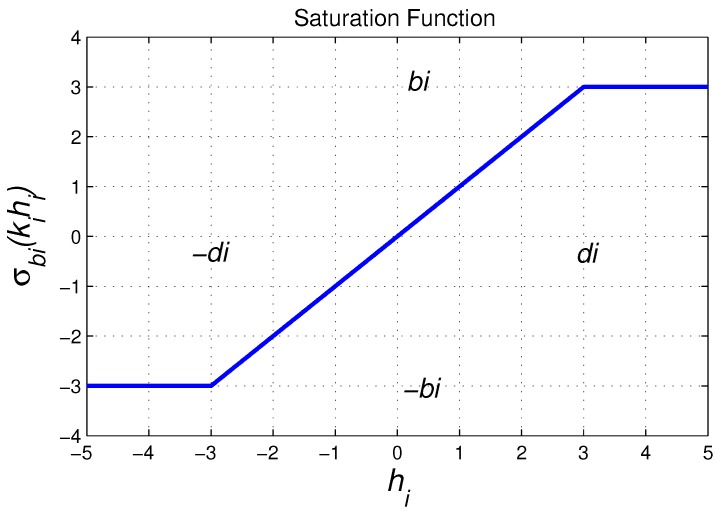
Saturated function.

Now, let $\sigma_{\bar{b_{i}}}$$\left(k_{i}h_{i}\right)$ be a saturation function ∀*i* = 1,2, and $\bar{b_{i}},k_{i}>0$, described in Figure 4 and defined as the next equation:

(14) $$\sigma_{\bar{b_{i}}}\left(k_{i}h_{i}\right)=\left\{\begin{array}{c} \bar{b_{i}},\;\;if\;k_{i}h_{i}>\bar{b_{i}},\\ k_{i}h_{i},\;if\;\left|k_{i}h_{i}\right|\leq \bar{b_{i}},\\ -\bar{b_{i}},\;if\;k_{i}h_{i}<-\bar{b_{i}}. \end{array}\right.$$

### 4.1. A Nonlinear PD Controller Based on Saturation Functions

According to Equations (13) and (14), we propose a nonlinear PD controller based on saturation functions as follows:

(15) $$u\left(t\right)=\sigma_{\bar{b_{1}}}\left[k_{1}e\left(t\right)\right]+\sigma_{\bar{b_{2}}}\left[k_{2}\frac{de(t)}{dt}\right].$$

The above equation can be represented as:

(16) $$u\left(t\right)=\sum_{i=1}^{2}u_{i},$$

where $u_{i}$=$\sigma_{\bar{b_{i}}}\left(k_{i}h_{i}\right)$ represents the saturation function, $h_{1}$ is the error and $h_{2}$ is the derivative error. Then, according to Equation (14), we have:

(17) $$u_{i}=\sigma_{\bar{b_{i}}}\left(k_{i}h_{i}\right)=\left\{\begin{array}{c} \bar{b_{i}}\;\;if\;k_{i}h_{i}>\bar{b_{i}},\\ k_{i}h_{i}\;if\;\left|k_{i}h_{i}\right|\leq \bar{b_{i}},\\ -\bar{b_{i}}\;if\;k_{i}h_{i}<-\bar{b_{i}}, \end{array}\right.$$

and, for the previous equation, we can rewrite it as:

(18) $$u_{i}=\left\{\begin{array}{c} sign\left(h_{i}\right)\bar{b_{i}},\;if\;\left|k_{i}h_{i}\right|>\bar{b_{i}},\\ \;k_{i}h_{i},\;if\;\left|k_{i}h_{i}\right|\leq \bar{b_{i}}. \end{array}\right.$$

In Equation (18), we can notice that the parameters tuning of the controller, which is described by Equation (15), are the gains $k_{i}$ and the saturation values $\bar{b_{i}}$, ∀*i*=1,2. Notice that the parameters tuning could be the saturation values $b_{i}$ and the interval of $h_{i}$ for which $u_{i}$ is lineal, thus we can choose the value of $h_{i}$ for which we want to saturate the law control. As a consequence, we are going to introduce a new parameter. For this, we consider the point of $h_{i}$ where $\left|u_{i}\right|=\bar{b_{i}}$; this is:

(19) $$\left|u_{i}\right|=\left|k_{i}h_{i}\right|=\bar{b_{i}}\;⟹\;\left|h_{i}\right|=\bar{b_{i}}/k_{i}.$$

Then, we define:

(20) $$d_{i}:=\bar{b_{i}}/k_{i}$$

as the point where:

(21) $$u_{i}=sign\left(h_{i}\right)\bar{b_{i}}\;\forall \;\left|h_{i}\right|>d_{i}.$$

According to Equations (20) and (21), we can represent the control law, which is given by Equation (18), in terms of the parameters $b_{i}$ and $d_{i}$ as follows:

(22) $$u_{i}=\left\{\begin{array}{c} sign\left(h_{i}\right)\bar{b_{i}},\;if\;\left|h_{i}\right|>d_{i},\\ \;\bar{b_{i}}d_{i}^{-1}h_{i},\;if\;\left|h_{i}\right|\leq d_{i}, \end{array}\right.$$

where the parameters tuning of the controller are $b_{i}$ and $d_{i}$, ∀*i*=1,2. In order that Equation (22) will be expressed in terms of $h_{i}$ when $\left|h_{i}\right|>d_{i}$, we have that:

(23) $$\;sign\left(h_{i}\right)\bar{b_{i}}=h_{i}sign\left(h_{i}\right)\bar{b_{i}}h_{i}^{-1}.$$

Then,

(24) $$\;sign\left(h_{i}\right)\bar{b_{i}}=\left|h_{i}\right|\bar{b_{i}}h_{i}^{-1}.$$

Considering that $\left|h_{i}\right|h_{i}^{-1}={\left|h_{i}\right|}^{-1}h_{i}$, then Equation (22) can be rewritten as:

(25) $$\;u_{i}=\left\{\begin{array}{c} \bar{b_{i}}{\left|h_{i}\right|}^{-1}h_{i}\;if\;\left|h_{i}\right|>d_{i},\\ \;\bar{b_{i}}d_{i}^{-1}h_{i}\;if\;\left|h_{i}\right|\leq d_{i}. \end{array}\right.$$

Finally, the control law defined by Equation (15) can be represented as:

(26) $$\;u\left(t\right)=u_{1}+u_{2}=k_{p}\left(e\right)e\left(t\right)+k_{d}\left(\dot{e}\right)\dot{e}\left(t\right),$$

with:

(27) $$\;k_{p}\left(e\right)=\left\{\begin{array}{c} \bar{b_{1}}{\left|e(t)\right|}^{-1}\;if\;\left|e(t)\right|>d_{1},\\ \;\bar{b_{1}}d_{1}^{-1}\;if\;\left|e(t)\right|\leq d_{1}, \end{array}\right.$$

(28) $$\;k_{d}\left(\dot{e}\right)=\left\{\begin{array}{c} \bar{b_{2}}{\left|\dot{e}\left(t\right)\right|}^{-1}\;if\;\left|\dot{e}\left(t\right)\right|>d_{2},\\ \;\bar{b_{2}}d_{2}^{-1}\;if\;\left|\dot{e}\left(t\right)\right|\leq d_{2}. \end{array}\right.$$

The advantage of this controller is that the maximum forces and torques are chosen by the parameters $\bar{b_{1}}$ and $\bar{b_{2}}$. Thus, we are sure that the actuators will not be damaged, but, in other cases, it is necessary that the forces and torques are slightly larger to correct the system error.

### 4.2. Stability Proof

Considering Equations (1) and (2), we propose the following control input:

(29) $$\tau =g\left(\eta \right)-J^{T}\left(\eta \right)\tau_{NPD}$$

with:

(30) $$\tau_{NPD}=\sigma_{\bar{b_{p}}}\left[k_{p}e\left(t\right)\right]+\sigma_{\bar{b_{d}}}\left[k_{d}\left(\dot{e}\left(t\right)\right)\right].$$

The equation before can be rewritten as:

(31) $$\tau_{NPD}=K_{p}\left(\cdot \right)e\left(t\right)+K_{d}\left(\cdot \right)\dot{e}\left(t\right),$$

with:

(32) $$\;K_{p}\left(\cdot \right)=\left[\begin{array}{c} \;K_{p_{1}}\left(\cdot \right)\;0\;\cdots \;0\\ \;0\;K_{p_{2}}\left(\cdot \right)\;\cdots \;0\\ \;\vdots \;\vdots \;\ddots \;\vdots \\ \;0\;0\;\cdots \;K_{p_{n}}\left(\cdot \right) \end{array}\right]>0,$$

(33) $$\;K_{d}\left(\cdot \right)=\left[\begin{array}{c} \;K_{d_{1}}\left(\cdot \right)\;0\;\cdots \;0\\ \;0\;K_{d_{2}}\left(\cdot \right)\;\cdots \;0\\ \;\vdots \;\vdots \;\ddots \;\vdots \\ \;0\;0\;\cdots \;K_{d_{n}}\left(\cdot \right) \end{array}\right]>0,$$

where:

(34) $$\;k_{p_{i}}=\left\{\begin{array}{c} {\bar{b}}_{p_{i}}{\left|e_{i}\left(t\right)\right|}^{-1}\;if\;\left|e_{i}\left(t\right)\right|>d_{p_{i}},\\ \;{\bar{b}}_{p_{i}}d_{p_{i}}^{-1}\;if\;\left|e_{i}\left(t\right)\right|\leq d_{p_{i}}, \end{array}\right.$$

(35) $$\;k_{d_{i}}=\left\{\begin{array}{c} {\bar{b}}_{d_{i}}{\left|\dot{e_{i}}\left(t\right)\right|}^{-1}\;if\;\left|\dot{e_{i}}\left(t\right)\right|>d_{d_{i}},\\ \;{\bar{b}}_{d_{i}}d_{d_{i}}^{-1}\;if\;\left|\dot{e_{i}}\left(t\right)\right|\leq d_{d_{i}}. \end{array}\right.$$

Considering the regulation case:

(36) $$\eta_{d}=cte\;⟹\;\dot{\eta }=0.$$

Assuming that:

(37) $$\;e=\eta -\eta_{d}\;⟹\;\dot{e}=\dot{\eta },$$

then Equation (29) can be rewritten as:

(38) $$\tau =g\left(\eta \right)-J^{T}\left(\eta \right)\left[K_{p}\left(e\right)e+K_{d}\left(\dot{e}\right)\dot{\eta }\right].$$

Now, we have the closed-loop system as follows:

(39) $$\;M\dot{\nu }+C\left(\nu \right)\nu +D\left(\nu \right)\nu =-J^{T}\left(\eta \right)\left[K_{p}\left(e\right)e+K_{d}\left(\dot{e}\right)\dot{\eta }\right].$$

Considering Equation (2), we have:

(40) $$\;M\dot{\nu }+C\left(\nu \right)\nu +D\left(\nu \right)\nu =-J^{T}\left(\eta \right)\left[K_{p}\left(e\right)e+K_{d}\left(\dot{e}\right)J\left(\eta \right)\nu \right].$$

We define $K_{dd}\left(\eta ,\dot{e}\right)=J^{T}\left(\eta \right)K_{d}\left(\dot{e}\right)J\left(\eta \right)$; then, the previous equation can be rewritten as:

(41) $$\;M\dot{\nu }+C\left(\nu \right)\nu +D\left(\nu \right)\nu =-J^{T}\left(\eta \right)K_{p}\left(e\right)e-K_{dd}\left(\eta ,\dot{e}\right)\nu .\;$$

Rewriting the previous equation, we have:

(42) $$\frac{d}{dt}\left[\begin{array}{c} e\\ \nu \end{array}\right]=$$

(43) $$\left[\begin{array}{c} \;\;\;\;\;\;J(\eta )\nu \\ \;M^{-1}\left[J^{T}\left(\eta \right)K_{p}\left(e\right)e-K_{dd}\left(\eta ,\dot{e}\right)\nu -C\left(\nu \right)\nu -D\left(\nu \right)\nu \right] \end{array}\right].$$

Observe that the unique origin is the equilibrium point. Now, we can propose the following Lyapunov function candidate:

(44) $$\;V\left(e,\nu \right)=\frac{1}{2}\nu^{T}M\nu +\int_{0}^{e}\xi^{T}K_{p}\left(\xi \right)d\xi .$$

According to Lemma 2 from [14], we have that V(e,ν) is globally positive definite and a radially unbounded function. The time derivative of the Lyapunov function candidate is:

(45) $$\dot{V}\left(e,\nu \right)=\nu^{T}M\dot{\nu }-e^{T}K_{p}\left(e\right)J\left(\eta \right)\nu .$$

By substituting the closed-loop Equation (41) into (45), we obtain:

(46) $$\dot{V}\left(e,\nu \right)=\nu^{T}J^{T}\left(\eta \right)K_{p}\left(e\right)e-\nu^{T}K_{dd}\left(\eta ,\dot{e}\right)\nu -\nu^{T}C\left(\nu \right)\nu -\nu^{T}D\left(\nu \right)\nu -e^{T}K_{p}\left(e\right)J\left(\eta \right)\nu .$$

Since $K_{p}\left(e\right)=K_{p}^{T}\left(e\right)$ and $C\left(\nu \right)=-C{\left(\nu \right)}^{T}$, Equation (46) becomes:

(47) $$\dot{V}\left(e,\nu \right)=-\nu^{T}\left[K_{dd}\left(\eta ,\dot{e}\right)+D\left(\nu \right)\right]\nu .$$

Assuming that D(ν)>0 and remembering that $K_{d}>0⟶K_{dd}>0$ and symmetric matrix, we then obtain that $\dot{V}\left(e,\nu \right)$ is a globally negative semidifinite function, and therefore we conclude stability of the equilibrium point. In order to prove asymptotic stability, we apply the Krasovskii–LaSalle’s theorem; then:

(48) $$\Omega =\left[\left[\begin{array}{c} \;e\\ \;\nu \end{array}\right]=\dot{V}\left(e,\nu \right)=0\right]=\left[\left[\begin{array}{c} \;e\\ \;\nu \end{array}\right]=\left[\begin{array}{c} \;e\\ \;0 \end{array}\right]\in R^{2n}\;\right].$$

Introducing ν=0 and $\dot{\nu }=0$ into Equation (41), we have e=0; therefore, we conclude that the equilibrium point is globally asymptotically stable.

## 5. Computer Vision Algorithm

### 5.1. Data Processing Chain

**Figure 5:**
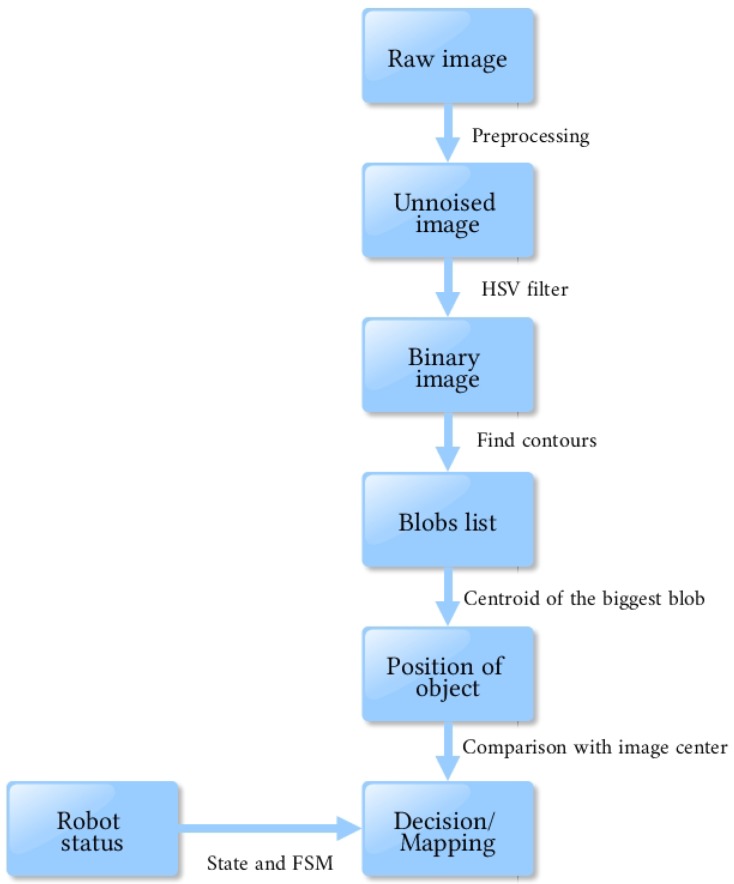
Data processing chain.

Processing an image requires many steps, each one narrowing the quantity of data until it gives what we need (see Figure 5).

Firstly, a raw image taken from the camera has to be unnoised thanks to a Gaussian blur. Then, only a pixel of wanted colors are kept, giving a binary image. At this step, the image gives a geometric representation of the interesting object, without any color considerations.

Secondly, the algorithm has to localize the object, in order to have coordinates, orientation, size and variables that can be easily manipulated. It detects all connected groups of pixels (called “blobs”), and returns their size and centroid position. The biggest is considered as the object that the robot has to follow.

Finally, depending on the status of the robot, it can decide to move or follow the detection of objects.

### 5.2. Image Preprocessing

Before trying to extract data from an image, we need to process some filters to remove most noise and uninteresting things. It usually involves spatial and temporal blur to fix noise variance and dynamic filters to fix noise mean.

Images provided by a camera have noise, which can corrupt computer vision algorithm. For instance, in order to detect features, we need to extract gradient, which can be distorted by noise variance. This kind of noise can be reduced by blur, especially Gaussian blur for Gaussian noise, which dilutes variance in neighboring pixels. However, this two-dimensional spatial blur should not be too strong, at the risk of producing diplopia. Moreover, the camera video has also a third dimension: time. A time blur along consecutive images could also reduce noise variance. However, it is rather effective on static video: mixing too many different images produces a ghost effect [15].

These previous filters are static, and they cannot change their parameters (which are calibrated for the hardware). We need dynamic filters to adapt automatically to the environment. Indeed, luminosity and contrast can change along time. A filter can keep the same luminosity, or change color distribution by analyzing and equalizing Hue histogram. As compensation, it increases noise variance [16]. The main issue is that we need to choose parameters depending on the environment. Completely independent methods exist, but they consume too much CPU resources, thus it could be useful to improve a single interesting image, just that is too much of a guzzler for autonomous navigation [17].

### 5.3. Extracting Interesting Data

The two main properties of objects in an image are color and shape. Color recognition is easy to implement, but not discriminating enough (different objects can have the same color). On the contrary, shape recognition is reliable, but not so easy to program.

We can combine advantages with these two methods: first, color recognition only keeps a little data corresponding to the right color, and then shape recognition processes these lightened data.

#### 5.3.1. HSV Filtering

**Figure 6:**
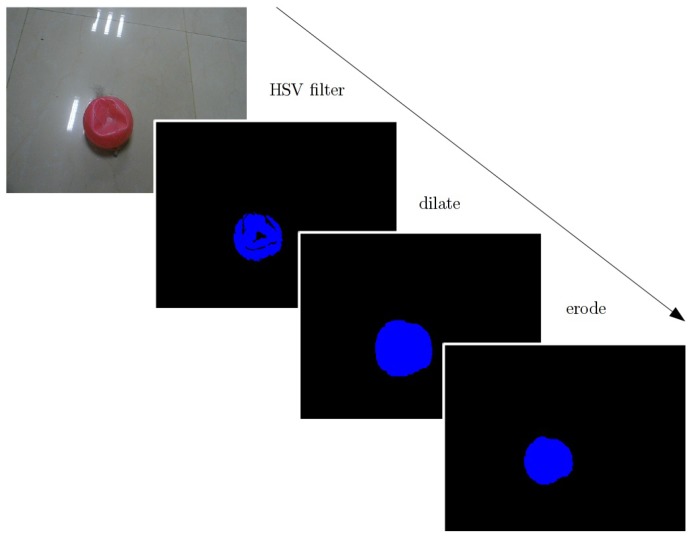
Extract interesting color with HSV filter.

The first step is HSV filtering. It keeps pixels whose “hue” corresponds to the right color, “saturation” and “value” above a threshold to eliminate gray and dark areas. The result is a binary image, in which white pixels are kept, and blue pixels are rejected. The issue is that many good pixels are rejected, which creates little holes. The solution is to “dilate” the image to recover these pixels, and “erode” to remove the border effect of dilate (see Figure 6).

#### 5.3.2. Blob Detection

**Figure 7:**
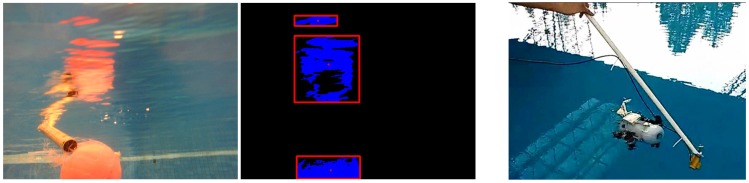
Segmentation to different blobs and the detection of another object.

Blob detection consists of linking adjacent pixels to create groups—each one corresponding to an object. This algorithm [18] is implemented in the “findContours” function of OpenCV. Then, each blob has its centroid computed, whose coordinates are understandable data for the navigation algorithm (see Figure 7).

### 5.4. Take Decision

Orders for motors are generated depending on the state of the FSM (Finite State Machine). Main states are:

- Remote control (no autonomous, the user sends orders).
- Stabilize (keep same position and posture).
- Go up and Go down (change only depth and stabilize).
- Explore (follow a planned path).
- Follow an object by the Raspberry Pi camera.

#### Ball Following through the Algorithm Vision

Blob detection gives the coordinates and size of each blob by giving HSV boundaries of interesting objects. It is required to know before the mission what the robot is looking for. The first step is to show to the robot an OPI (Object of Potential Interest) and manually select HSV boundaries until it is well detected. It must be done underwater because colors are not the same in the laboratory (Figure 6 and Figure 7 show pictures of the same OPI, outside and inside water). On the other hand, objects that avoid its reflection on the surface due to the surrounding light should be considered to obtain a better detection of the object. In addition, another object was detected as shown in Figure 7. However, the detection of the object was fine while the sun is neglected on the surface, due to the yellow color of this object. Therefore, the detection of the objects is good until they are not close to the surface when their reflection appears or they are confused with others of the same color.

Then, the robot is able to find the coordinate ($x_{opi}$, $y_{opi}$) of the biggest object seen by its camera, which it will try to follow. Due to the shape of AR2D2, a simple proportional law control is enough. By comparing to the center ($x_{c}$, $y_{c}$) of the image, and its size to a threshold size thresh, we obtain this kind of control law:

(49) $$\left[\begin{array}{c} mot_{1}\\ mot_{2}\\ mot_{3}\\ mot_{4} \end{array}\right]=\left[\begin{array}{c} +K_{py}\\ +K_{py}\\ +K_{px}\\ -K_{px} \end{array}\right]*\left[\begin{array}{c} y_{opi}-y_{c}\\ y_{opi}-y_{c}\\ x_{opi}-x_{c}\\ x_{opi}-x_{c} \end{array}\right]+K_{size}*\left[\begin{array}{c} 0\\ 0\\ size_{tresh}-size\\ size_{tresh}-size \end{array}\right].$$

**Figure 8:**
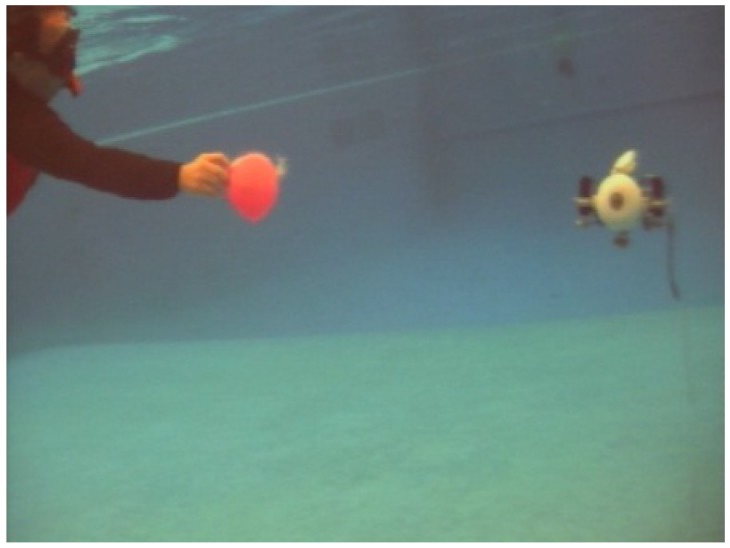
Ball following test into the swimming pool.

$K_{px}$ and $K_{py}$ try to minimize horizontal and vertical error of the OPI’s centroid compared to the center of the image. Meanwhile, $K_{size}$ keeps the robot to a good distance from the object (here, the size gives an approximation of distance). In Figure 8, we can observe an experimental test of the detection of the ball.

## 6. Simulation and Results

Matlab Simulink (R2016a, MathWorks) has been used to apply the control law chosen in order to stabilize the surge and heave movements on the dynamic model equations.

**Figure 9:**
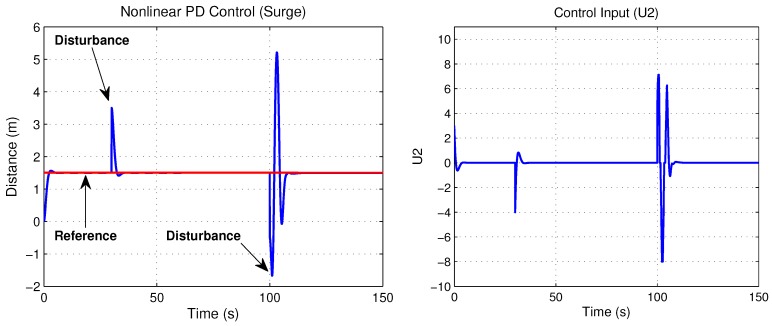
Surge movement and its control input U2.

**Figure 10:**
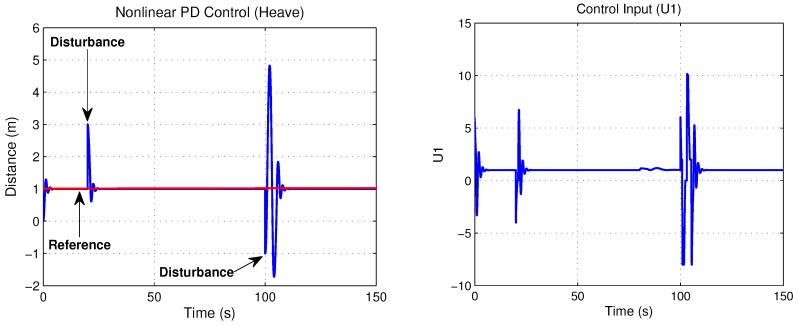
Heave movement and its control input U1.

Simulation results are presented in order to observe the performance of the translation movements by the proposed control law. Nonlinear PD control was tuned to observe the best behavior versus disturbances, where the proportional and derivative gains on the saturation functions relative to surge and heave control, belong to values; b=+/−4 and d=+/−2, thus the time evolution of the “*x*” vehicle’s position is shown in Figure 9, while its “*z*” position of the closed-loop system are plotted in Figure 10. Observe that the state positions (x,z) are externally perturbed and the nonlinear PD control is again able to stabilize the engine position. The initial conditions used for this simulation are $x\left(0\right)=0,z\left(0\right)=0,\dot{x}\left(0\right)=0,\dot{z}\left(0\right)=0$ and the desired values are $x_{d}=1.5$ and $z_{d}=1$.

Control input U1 is directly applied in vertical forces and control input U2 is applied in horizontal forces. Figure 9 and Figure 10 show both control inputs. Observe that control input U1 converges to the weight value $f_{W}$ = 1, whilst that control input U2 converges to zero, due to horizontal forces’ combination.

**Figure 11:**
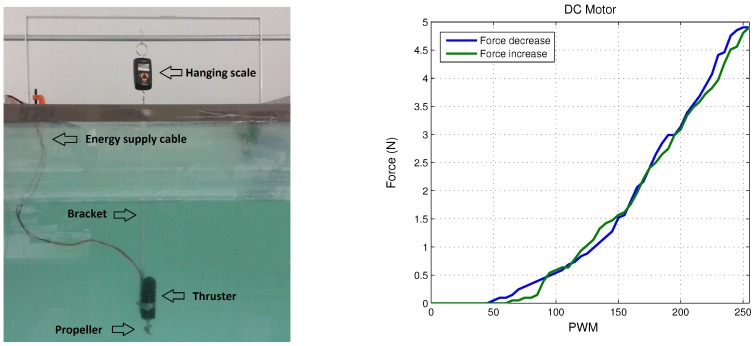
Thruster test.

In order to characterize the thrusters, we did some tests in the fish tank, considering the reaction of the thruster on the *z*-axis through a PWM signal (Pulse Width Modulation) for the maneuverability index (see Figure 11). Therefore, F = (PWM/50) − 1 is the linear equation obtained by the adjustment of the curve. On the other hand, in recent works, such as in [19], it is important to consider a greater knowledge of the dynamic of all the thrusters to obtain the best performance and efficiency with respect to the maneuvers of the vehicle in control applications. In this case, the Micro AUV has the layout configuration of the thrusters near the center of gravity to obtain better maneuverability.

**Figure 12:**
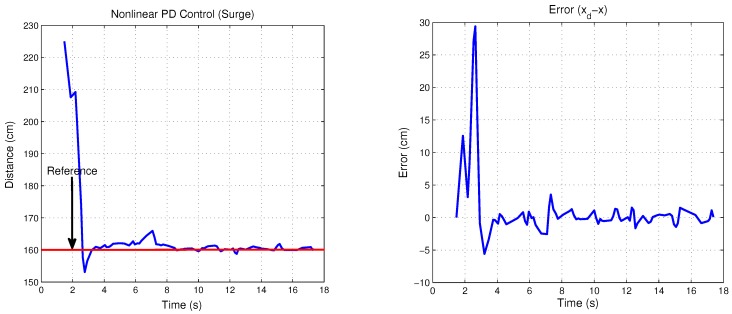
Experimental surge movement and its signal of error in regulation.

**Figure 13:**
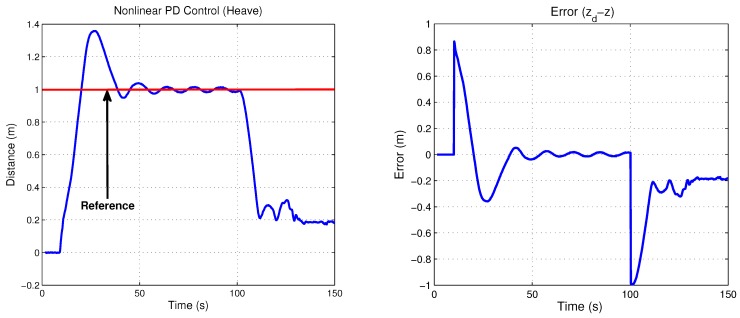
Experimental heave movement and its signal of error in regulation.

**Figure 14:**
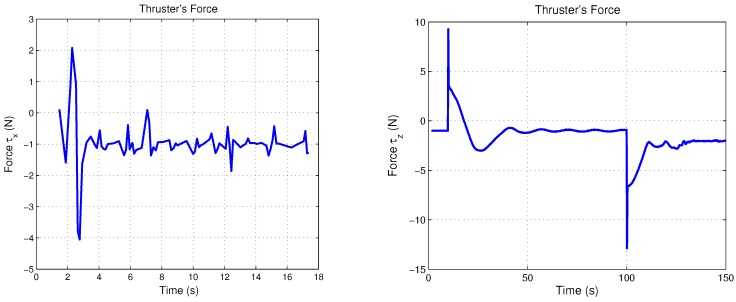
Experimental reaction of the torques on the *x*- and *z*-axes.

Figure 12 and Figure 13 show the experimental results relative to surge and heave movements and its respective error signal in the regulation, while the torques of the thrusters on both axes are depicted in Figure 14. Notice that the control law proposed presents a good performance. The gains tuning was fixed in order to get the best behaviour of the micro submarine exposed to extern disturbs. Concerning the stability improvement, it is possible to fix the gains tuning with more accuracy, considering the controller characteristic, being able to saturate the control input and protect the actuators.

The ball followed a random path (it is not a stabilization test) with random velocity. The robot managed to follow it, even when the object goes out of image. Indeed, inertia of the robot into water preserves its movement, making the robot able to find the OPI again.

## 7. Conclusions

The development of this prototype was motivated by the need for having an autonomous small vehicle for operation in closed environments. We developed and presented the embedded control system of the AR2D2 Micro AUV robot. In this initial work, we have considered the problem of set-point regulation on heave and surge movement. The vehicle was designed with the aim of having a small platform able to make complex maneuvers; to this end, the dynamic decoupling of surge and sway turned to be a key issue. In turn, the navigation embedded system was reduced as much as possible. On one hand, one is restricted to work in closed volumes, and, on the other hand, we are looking for cooperative tasks as the major goal of our future research work [20]. In this paper, we have used a typical nonlinear controller with saturation functions, thus the closed loop stability was demonstrated on the basis of Lyapunov’s theory. The desired behavior was validated by Matlab’s simulation and experiments. On the other side, this vehicle performs real-time embedded image processing in order to recognize and follow an object of interest. The results show a good ability to do it, without losing the OPI. In the future, we will implement another control strategy aiming to improve the performances along yaw angle and regulation in *x*, in order to be close to the object for further inspection. Introducing some filters along time can also improve behavior of the robot, and stabilize recognition of object (by processing sequential video images instead of single pictures). Moreover, we will conduct experiments in a natural environment with less visibility.
